# From evaluation to discharge: a cross-sectional study of therapist satisfaction with session duration in outpatient physical therapy

**DOI:** 10.7717/peerj.21394

**Published:** 2026-06-08

**Authors:** Saad A. Alhammad, Omar Khalid Almuhanna, Muteb Safar Aldosari, Abdulaziz Riyadh Aljumaah

**Affiliations:** Department of Rehabilitation Health Sciences, College of Applied Medical Sciences, King Saud University, Riyadh, Saudi Arabia

**Keywords:** Physical therapy, Schedules and appointments, Saudi Arabia, Outpatient clinics, Session duration, Satisfaction, Healthcare policies

## Abstract

**Background:**

Physical therapy (PT) session duration influences satisfaction, therapeutic effectiveness, and workload. Although prior studies examined satisfaction and outcomes, limited research has described how session duration is structured across phases of outpatient care. In Saudi Arabia, where rehabilitation services are expanding, no standard guidance exists for outpatient session length. This study explored current durations of evaluation, follow-up, and discharge sessions in outpatient PT across diverse patient populations, and examined therapists’ satisfaction and perspectives on adequacy and optimal duration.

**Methods:**

A cross-sectional survey was distributed to PTs practicing in outpatient and homecare settings across Saudi Arabia. The questionnaire collected demographic data, current session durations, satisfaction levels, and preferences for more or less time. Descriptive statistics and cross-tabulations examined variation by session type, patient population, daily caseload, and overbooking.

**Results:**

A total of 434 PTs participated. The most commonly reported durations were 30–40 and 15–25 min for evaluation (36%, 35%), follow-up (45%, 25%), and discharge sessions (39%, 33%). Duration varied by patient population; for example, 45–55-min evaluation sessions were reported by 41% of pediatric therapists *vs*. 18% of musculoskeletal therapists. Satisfaction increased with longer durations (evaluation: 48% at 15–25 min *vs*. 86% at ≥60 min). Among therapists practicing overbooking, satisfaction ranged from 47% (15–25 min) to 76% (≥60 min), compared with 55% to 85% among those not overbooking. Among therapists preferring longer sessions, optimal durations clustered at 30–40 and 45–55 min (evaluation: 46%, 35%; follow-up: 40%, 37%; discharge: 42%, 36%). Among those preferring shorter sessions, 15–25 min was consistently selected (evaluation: 71%; follow-up: 47%; discharge: 59%). Therapists preferring longer sessions most often cited the need for more time for physical examination, exercise prescription/monitoring, and patient education (evaluation: 78%, 53%, 64%; follow-up: 38%, 78%, 63%; discharge: 51%, 49%, 69%), whereas those preferring shorter sessions primarily reported avoiding wasted time (94%, 81%, 71%).

**Discussion:**

This study provides the first national description of outpatient PT session duration and therapist satisfaction in Saudi Arabia. Preferences were shaped by clinical context, caseload, and scheduling practices. Within its descriptive scope, these distributions may serve as practical scheduling benchmarks, informing default duration ranges by visit phase and patient population and guiding review of overbooking and caseload targets while supporting future research on clinical outcomes.

## Introduction

Physical therapy (PT) plays a central role in the rehabilitation of individuals with musculoskeletal, neurological, pediatric, and cardiopulmonary conditions, aiming to restore movement, reduce disability, and improve quality of life (American Physical Therapy Association, 2025a; World Physiotherapy, 2023; Australian Physiotherapy Association, 2025). Physical Therapists (PTs) are responsible for evaluating patients, developing care plans, and implementing individualized treatment strategies that rely on comprehensive assessments, clinical reasoning, and patient education (World Physiotherapy, 2023; Australian Physiotherapy Association, 2025). A key element influencing these interventions is the amount of time allocated for clinical sessions, as outpatient physical therapy encompasses diverse clinical areas with differing assessment and treatment demands that may shape expectations regarding session duration. Several studies have noted that time availability influences patient satisfaction, communication, and the quality of therapist-patient interactions in outpatient rehabilitation settings (Waters et al., 2016; O’Keeffe et al., 2015; Medina-Mirapeix et al., 2013).

In other healthcare settings, consultation length has been linked to emotional satisfaction, as Ogden et al. (2004) found a significant correlation between a desire for more time and satisfaction with the emotional content of the consultation. Longer consultations have also been linked to better recognition of psychosocial issues (Freeman et al., 2002) and are even used as a proxy for quality of care in UK health systems (Elmore et al., 2016). These findings reinforce the broader idea that time is not merely administrative but functionally tied to the quality of care.

Although peer-reviewed international benchmarks for outpatient physical therapy session duration are limited, operational guidance and service specifications indicate common structuring in practice: evaluation visits are typically longer than follow-up visits. For example, APTA lists typical face-to-face times of ~20, 30, and 45 min for low-, moderate-, and high-complexity evaluations, and several NHS services advise first appointments of ~45 min with follow-ups of ~20–30 min (American Physical Therapy Association, 2025b; Dorset HealthCare University NHS Foundation Trust, 2025; HIOW Healthcare (NHS), 2025). While templates vary and no universal “optimal” duration exists, chosen session durations shape appointment templates and clinician capacity, with implications for staffing, scheduling, and access to outpatient PT.

Perceptions of time sufficiency have been reported as contributing to patient satisfaction in physical therapy, particularly when patients feel their sessions are not rushed or interrupted (Waters et al., 2016; Hush, Cameron & Mackey, 2011; Rossettini et al., 2020). As Hush, Cameron & Mackey (2011) observed, patients ‘need to feel they have had adequate time with the therapist and not feel rushed through an appointment’. Additionally, Issa et al. (2013) found that patients reporting greater satisfaction had significantly longer average PT session durations. While much of the existing literature has focused on patient satisfaction and perceived quality of care, considerably less attention has been given to therapists’ perspectives on whether allocated session time is adequate to perform essential clinical tasks. The present study therefore focuses specifically on clinicians’ expectations and judgments regarding session duration, while recognizing that session duration inherently affects both therapists and patients within the therapeutic encounter.

This gap is particularly relevant in Saudi Arabia, where rehabilitation services are expanding under Vision 2030 (Saudi Vision 2030, 2025), but no standardized guidelines exist for outpatient session duration. While prior studies in Saudi Arabia have explored patient satisfaction and appointment scheduling in the outpatient setting (Algudairi et al., 2018; Ali et al., 2023; Alhammad et al., 2025), the question of how much time is allocated per session, and whether that time is sufficient remains largely unaddressed.

However, we were unable to identify studies that report session duration by visit phase (evaluation, follow-up, discharge) together with therapists’ satisfaction, perceived adequacy, and preferred/optimal duration, and, to our knowledge, no such description exists for outpatient PT in Saudi Arabia. This study aims to assess the current duration of evaluation, follow-up, and discharge sessions in outpatient physical therapy, and to explore therapists’ satisfaction and perspectives on adequacy and optimal duration.

## Materials and Methods

This observational cross-sectional study used a self-administered electronic questionnaire to collect data from PTs practicing in outpatient departments and homecare services across Saudi Arabia. Eligible participants were currently working in clinical outpatient or homecare settings. PTs working exclusively in inpatient care or administrative roles were excluded.

The questionnaire was distributed and was only available for between February 25 and May 26, 2024, *via* social media platforms (X, Instagram, LinkedIn, and WhatsApp) and in-person visits to hospitals and PT centers. After the initial invitation, we sent 2–3 reminders at 7 and 14 days through the same used social media channel, targeting non-responders only. Ethical approval was granted by the King Saud University Institutional Review Board (KSU-IRB#E-24-8553). Participation was voluntary and anonymous, and all participants provided informed consent electronically. A comprehensive sampling frame of outpatient PTs was not available; therefore, convenience recruitment was used to maximize reach across sectors (Governmental and Private sectors) and regions of Saudi Arabia (Riyadh, Makkah, Eastern Province, Madinah, Asir, Tabuk, Al-Qassim, Hail, Jazan, Najran, Al-Bahah, Al-Jawf, and Northern Borders). No formal *a priori* sample size calculation was performed, as the study was designed to be exploratory and descriptive. The questionnaire was developed for this study and disseminated to five experienced physical therapists from different practice areas; their feedback on clarity, relevance, and wording was incorporated, and the questionnaire was modified accordingly. The average completion time was 6–8 min. All questionnaire items were written in English. The questionnaire consisted of two main sections. The first section gathered demographic and practice-related information, including age, sex, nationality, years of experience, primary clinical setting, primary patient population, whether the clinic used overbooking practices, and the number of patients seen per day (caseload). The second section focused on session duration. Participants were asked to report their current session durations for evaluation, follow-up, and discharge sessions. For each session type, participants were asked whether they were satisfied with the current duration, and whether they considered it optimal, wanted more time, or wanted less time. Those who indicated a preference for more or less time were then asked to specify what they considered the optimal session duration and to select the main reasons for their preference.

All analyses were conducted in Microsoft Excel for Microsoft 365 (Version 2510 Build 16.0.19328.20144) on Windows 11. No missing data were observed because responses to key items were mandatory and completeness checks were enforced before submission. Because study variables were collected as categorical ranges (nominal/ordinal), counts (n) and percentages (%) are reported, and the most frequent (modal) categories are highlighted where informative. Means/SD and medians/IQRs were not computed because the categorical bins preclude valid averaging. Cross-tabulations were used to compare categories and explore patterns in therapist satisfaction and duration preferences across session type, daily caseload groups, overbooking status, and primary patient population. No hypothesis tests or regression models were prespecified or performed, consistent with the descriptive scope of the study; findings are presented as descriptive patterns among respondents.

## Results

### Participant characteristics

**Figure 1:**
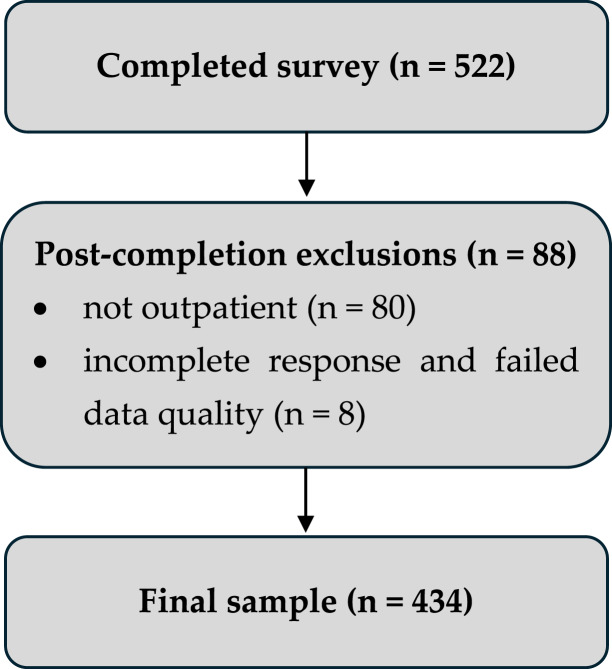
Participant flow diagram. Flow of participants from survey completion to final sample (Feb 25–May 26, 2024). Boxes show completed, post-completion exclusions with reasons, and the final sample. Recruitment occurred *via* social media platforms and in-person visits to hospitals and PT centers; total reach not ascertainable.

The present study was conducted between February 25 and May 26, 2024. A total of 522 respondents completed the survey; after excluding 88 post-completion responses (non-outpatient setting or incomplete/failed quality), the final sample included in analysis was *n* = 434 Fig. 1. Participants practiced in outpatient and homecare settings across Saudi Arabia. The sample was predominantly female (56%), with most participants aged 20–29 years (69%). Most were Saudi nationals (93%) and held an undergraduate degree as their highest qualification (84%). Regarding clinical experience, 76% reported 1–5 years. Most PTs worked in private sector facilities (59%) and were based primarily in the central region (55%). The majority (67%) specialized in musculoskeletal care. Regarding caseload, nearly half (50%) reported seeing 5–10 patients per day. All participant characteristics are shown in Table 1.

**Table 1:** Participant characteristics.

	Number (*n*)	Percentage (%)
Gender		
Male	193	44
Female	241	56
Age (years)		
20–29	301	69
30–39	114	26
≥40	19	4
Nationality		
Saudi	405	93
Non-Saudi	29	7
Level of education		
Undergraduate	363	84
Postgraduate	71	16
Years of experience (years)		
1–5	331	76
6–10	59	14
≥11	44	10
Work setting		
Private^1^	258	59
Public^2^	133	31
Other^3^	43	10
Employment region		
Central	240	55
Western	98	23
Eastern	47	11
Southern	35	8
Northern	17	4
Primary patient population seen by the therapist		
Musculoskeletal	291	67
Pediatric	54	12
Sports	45	10
Neurology	30	7
Other^4^	17	4
Number of patients seen daily		
1–5	110	25
6–10	217	50
11–15	71	17
≥16	36	8
Overbooking^5^		
Yes	230	53
No	204	47

**Notes:**

1 Private: outpatient services in private hospitals or private clinics.

2 Public: outpatient services in public hospitals or public clinics.

3 Other (work setting): includes homecare, daycare centers, educational institutions, and mixed public-private settings.

4 Other (subspecialties): includes general or mixed practice, cardiopulmonary and vascular, lymphedema, oncology, vestibular, and women’s health.

5 Overbooking: treating more than one patient in the same timeframe (group sessions are not included).

### Current session durations, satisfaction, and preferences

The most commonly reported evaluation session durations were 30–40 min (36%) and 15–25 min (35%). Satisfaction was highest in the 45–55 min group (82%) and lowest in the 15–25 min group (48%). Preference for more time was most frequent among therapists with 15–25 min sessions (42%). While the 45–55 min group had the highest proportion reporting their session as optimal (79%). For follow-up sessions, the most commonly reported duration was 30–40 min (45%), followed by 15–25 min (25%) and 45–55 min (25%). Satisfaction was highest in the 45–55 min group (76%) and lowest in the 15–25 min group (51%). Preference for more time was most frequent in the 15–25 min group (39%). While the 45–55 min group had the highest proportion reporting their session as optimal (81%). Regarding discharge sessions, 30–40 min (39%) and 15–25 min (33%) were the most reported durations. Satisfaction was highest in the 45–55 min group (78%) and lowest in the 60+ min group (59%). Preference for more time was most common in the 15–25 min group (20%), and the highest proportion reporting optimal duration was in the 30–40 min group (83%). All corresponding values are detailed in Table 2.

**Table 2:** Current physical therapy session durations, satisfaction levels, and time preferences among physical therapists.

Current session duration	*n* (% of total)	Satisfaction levels (% within each duration group)			Time preference (% within each duration group)		
		Satisfied	Dissatisfied	Neutral	Duration is optimal	Want more time	Want less time
Evaluation							
15–25 min	151 (35%)	73 (48%)	37 (25%)	41 (27%)	84 (56%)	64 (42%)	3 (2%)
30–40 min	158 (36%)	108 (69%)	15 (9%)	35 (22%)	119 (75%)	31 (20%)	8 (5%)
45–55 min	97 (22%)	80 (82%)	4 (4%)	13 (13%)	77 (79%)	15 (16%)	5 (5%)
60+ min	28 (6%)	24 (86%)	1 (3%)	3 (11%)	25 (89%)	2 (7%)	1 (4%)
Follow-up							
15–25 min	107 (25%)	55 (51%)	38 (36%)	14 (13%)	59 (55%)	42 (39%)	6 (6%)
30–40 min	197 (45%)	139 (70%)	19 (10%)	39 (20%)	150 (76%)	37 (19%)	10 (5%)
45–55 min	108 (25%)	82 (76%)	8 (7%)	18 (17%)	87 (81%)	10 (9%)	11 (10%)
60+ min	22 (5%)	15 (68%)	4 (18%)	3 (14%)	13 (59%)	4 (18%)	5 (23%)
Discharge							
15–25 min	143 (33%)	92 (64%)	20 (14%)	31 (22%)	106 (74%)	28 (20%)	9 (6%)
30–40 min	168 (39%)	114 (68%)	16 (9%)	38 (23%)	139 (83%)	17 (10%)	12 (7%)
45–55 min	94 (22%)	73 (78%)	7 (7%)	14 (15%)	77 (82%)	11 (12%)	6 (6%)
60+ min	29 (7%)	17 (59%)	5 (17%)	7 (24%)	19 (66%)	3 (10%)	7 (24%)

**Note:**

“Current Session Duration” denotes the session type (evaluation, follow-up, or discharge) and the reported duration category. The *n* (% of total) column presents the number and percentage of the full analytic sample (*n* = 434) in each duration category, reported separately for evaluation, follow-up, and discharge sessions. The Satisfaction Levels and Time Preference columns show the number and row percentage within each session type–duration category; accordingly, percentages within each row sum to 100% (subject to rounding).

### Variation in current session durations by patient population

**Figure 2:**
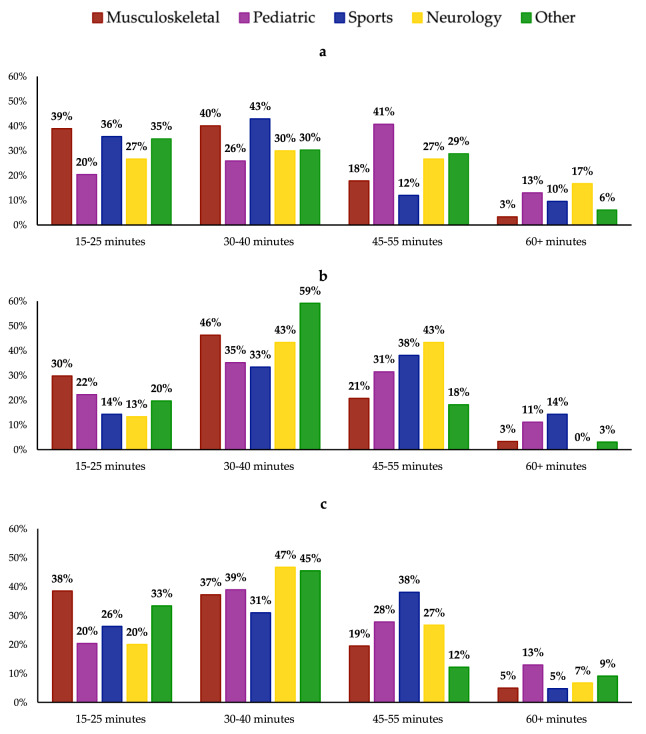
Current session durations across different patient populations. Reported session durations across evaluation, follow-up, and discharge sessions, stratified by the therapist’s primary patient population. (A)–(C) represent evaluation, follow-up, and discharge sessions. Bars show the percentage of therapists within each patient population selecting each session-duration interval. Duration categories were 15–25, 30–40, 45–55, and 60+ min. Percentages were calculated within each patient population group. Because group sizes differed across populations (MSK *n* = 291, Pediatrics *n* = 54, Neurology *n* = 30, Sports *n* = 45, Other *n* = 17), direct comparisons should be interpreted cautiously.

Session durations varied across patient populations and session types. Musculoskeletal therapists most frequently reported 30–40 min and 15–25 min for evaluation (40% and 39%), follow-up (46% and 30%), and discharge sessions (37% and 38%). Pediatric therapists most frequently reported 45–55 min and 30–40 min for evaluation (41% and 26%), follow-up (31% and 35%), and discharge sessions (28% and 39%). Neurology therapists most frequently reported 30–40 min and 45–55 min for evaluation (30% and 27%), follow-up (43% and 43%), and discharge sessions (47% and 27%). Sports therapists most frequently reported 30–40 min and 15–25 min for evaluation (43% and 36%) and 30–40 min and 45–55 min for follow-up and discharge sessions (follow-up: 33% and 38%; discharge: 31% and 38%). Therapists treating other populations most frequently reported 30–40 min and 15–25 min for evaluation (30% and 35%), follow-up (59% and 20%), and discharge sessions (45% and 33%). All data are illustrated in Fig. 2. Across all patient populations and session types, satisfaction was over 50%.

### Session duration satisfaction patterns by overbooking practice

**Figure 3:**
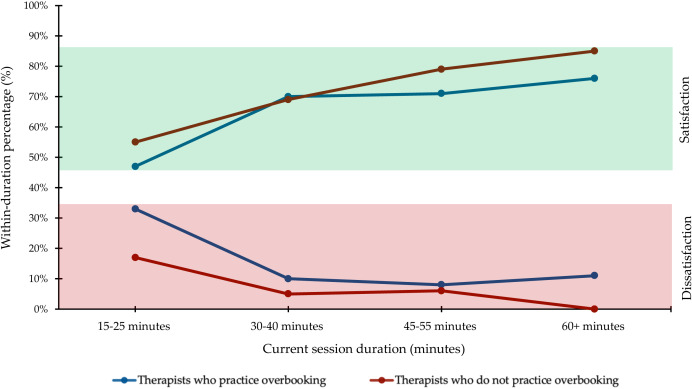
Session duration satisfaction patterns by overbooking practice. Satisfaction and dissatisfaction with current session duration by overbooking practice. The upper shaded panel shows the percentage of respondents satisfied with their current session duration, and the lower shaded panel shows the percentage dissatisfied. Percentages are calculated within each session-duration and overbooking group. Overbooking was defined as treating more than one patient in the same timeframe, excluding group sessions. Duration categories were 15–25, 30–40, 45–55, and 60+ min.

Session satisfaction levels differed across therapists based on whether they practiced overbooking, treating more than one patient in the same timeframe. Data from evaluation, follow-up, and discharge sessions were combined to provide an overall view of the relationship between overbooking practices and satisfaction across outpatient care. Among those who reported practicing overbooking, satisfaction rates were 47% for sessions lasting 15–25 min, 70% for 30–40 min, 71% for 45–55 min, and 76% for 60+ min. Corresponding dissatisfaction rates were 33%, 10%, 8%, and 11%, respectively. On the other hand, therapists who did not practice overbooking consistently reported higher satisfaction. Satisfaction levels were 55% for sessions of 15–25 min, 69% for 30–40 min, 79% for 45–55 min, and 85% for 60+ min. Dissatisfaction rates for this group were lower, reported at 17%, 5%, 6%, and 0%, respectively. All data are illustrated in Fig. 3.

### Session duration satisfaction patterns by daily caseload

**Figure 4:**
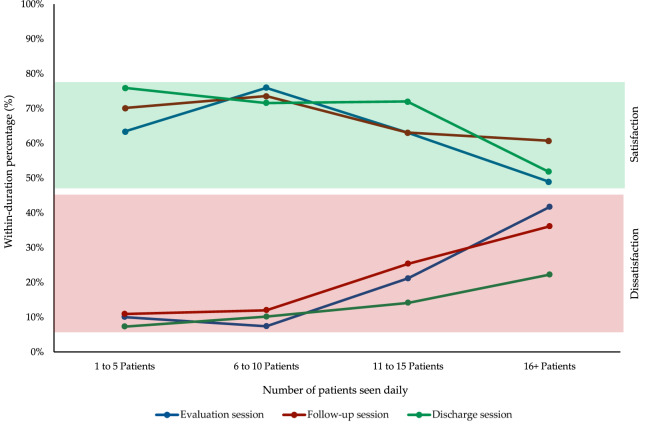
Session duration satisfaction patterns by number of patients seen daily. Satisfaction and dissatisfaction with current session duration by number of patients seen daily. The upper shaded panel shows the percentage of respondents satisfied with their current session duration, and the lower shaded panel shows the percentage dissatisfied. Lines represent evaluation, follow-up, and discharge sessions. Percentages were calculated within each daily patient-volume and session-type group. Daily patient-volume categories were 1–5, 6–10, 11–15, and 16+ patients.

Satisfaction with session durations varied notably by therapists’ daily caseloads, number of patients seen daily. For those seeing 1–5 patients per day, satisfaction rates were 61% for evaluation, 67% for follow-up, and 73% for discharge sessions, while dissatisfaction rates were 10%, 11%, and 7%, respectively. Among therapists seeing 6–10 patients daily, satisfaction rates increased to 73% for evaluation, 71% for follow-up, and 69% for discharge sessions, with corresponding dissatisfaction rates of 7%, 12%, and 10%. In the group treating 11–15 patients per day, satisfaction was reported at 61% for evaluation, 61% for follow-up, and 69% for discharge sessions. Meanwhile, dissatisfaction rates rose to 21%, 25%, and 14%, respectively. Finally, for those seeing 16 or more patients daily, satisfaction dropped to 47% for evaluation, 58% for follow-up, and 50% for discharge sessions. Dissatisfaction rates were highest in this group, at 42%, 36%, and 22%, respectively. All data are illustrated in Fig. 4.

### Preferred optimal durations

**Figure 5:**
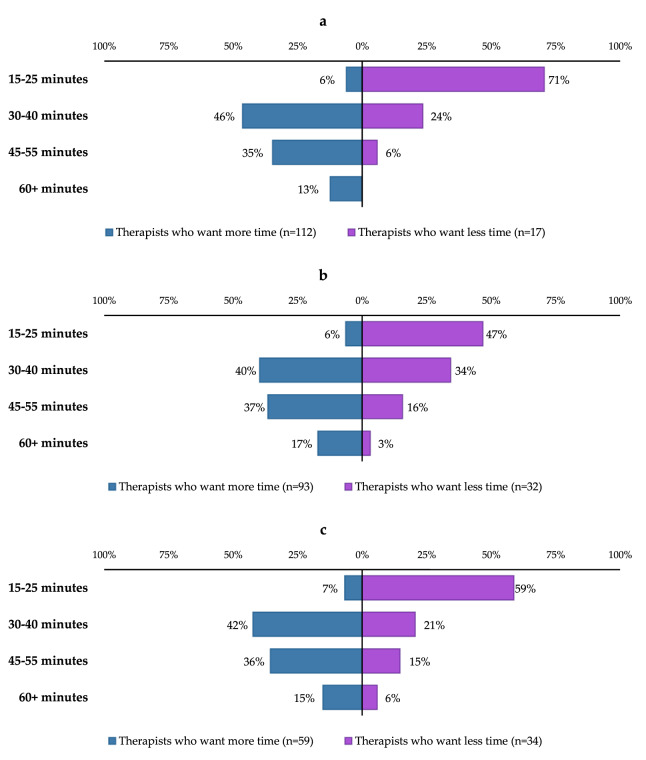
Preferred session durations among therapists wanting more or less time. Preferred session durations among therapists who reported wanting more or less time. (A)–(C) correspond to evaluation, follow-up, and discharge sessions, respectively. Within each panel, bars extending to the left represent therapists who wanted more time, whereas bars extending to the right represent therapists who wanted less time. Bars show the percentage of respondents within each preference group selecting each duration category as optimal; thus, percentages sum to 100% separately within the “want more time” and “want less time” groups for each session type. Duration categories were 15–25, 30–40, 45–55, and 60+ min (Fig. 5). Preferred session durations among therapists who reported wanting more or less time. (A–C) correspond to evaluation, follow-up, and discharge sessions, respectively. Within each panel, bars extending to the left represent therapists who wanted more time, whereas bars extending to the right represent therapists who wanted less time. Bars show the percentage of respondents within each preference group selecting each duration category as optimal; thus, percentages sum to 100% separately within the “want more time” and “want less time” groups for each session type. Duration categories were 15–25, 30–40, 45–55, and 60+ min.

Among therapists who wanted more time, the most frequently selected optimal durations across all session types were 30–40 min and 45–55 min. For evaluation sessions (*n* = 112), 46% chose 30–40 min and 35% selected 45–55 min. In follow-up sessions (*n* = 93), 40% preferred 30–40 min and 37% chose 45–55 min. Similarly, in discharge sessions (*n* = 59), 42% identified 30–40 min as optimal and 36% selected 45–55 min. Among therapists who wanted less time, the most preferred duration across session types was consistently 15–25 min. For evaluation sessions (*n* = 17), 71% selected 15–25 min as optimal. In follow-up sessions (*n* = 32), 47% preferred 15–25 min, and for discharge sessions (*n* = 34), 59% chose 15–25 min. Preferences for 30–40 min followed, with 24%, 34%, and 21% selecting it for evaluation, follow-up, and discharge sessions, respectively. All data are illustrated in Fig. 5.

### Reasons for time preferences

Among therapists who preferred longer evaluation sessions (*n* = 112), the most frequently reported reasons were the need for more time to conduct a physical examination (78%), obtain a thorough patient history (71%), and provide adequate patient education (64%). Additional reasons included allowing more time to explain the treatment plan (56%), prescribe or monitor exercises (53%), provide passive treatments (46%), complete documentation (44%), prepare the clinic or perform cleanup (32%), allow patients to change clothes (31%), and allow time for warm-up (21%). A few therapists (3%) reported other reasons, such as addressing family questions, helping patients acclimate to the clinical environment, or managing pediatric cases requiring extended interaction. On the other hand, therapists who preferred shorter evaluation sessions (*n* = 17) most frequently reported avoiding wasted time for both therapist and patient (94%), followed by the desire to see more patients (35%) and reduce working hours (12%).

Moreover, for therapists who preferred longer follow-up sessions (*n* = 93), the most commonly selected reasons were the need for more time to prescribe or monitor exercises (78%), provide passive treatments (73%), and deliver patient education (63%). Other reasons included explaining the treatment plan (49%), allowing patients time to warm up (49%), completing documentation (44%), conducting a physical examination (38%), allowing time to change clothes (37%), preparing the clinic or performing cleanup (37%), and obtaining patient history (27%). Conversely, therapists who preferred shorter follow-up sessions (*n* = 32) most frequently reported avoiding wasted time (81%), followed by a desire to see more patients (28%) and reduce working hours (6%). Others (9%) mentioned improving session quality, taking sufficient rest between sessions, and adjusting duration to better suit pediatric patients’ endurance.

**Figure 6:**
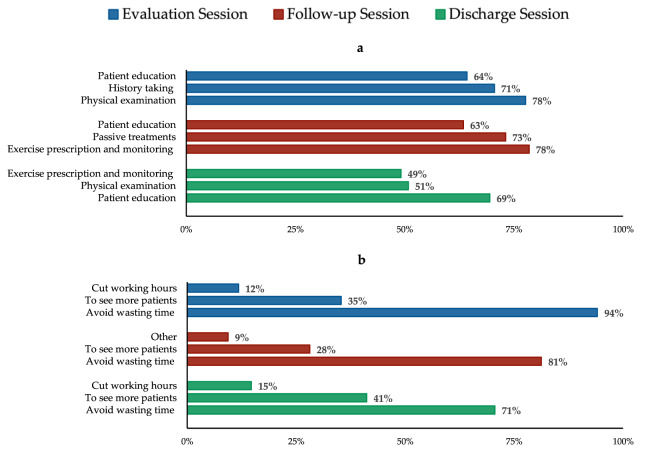
Reasons physical therapists want more or less time for their current session duration. Reasons for preferring more or less time across evaluation, follow-up, and discharge sessions. (A) and (B) show responses from therapists who preferred more time and less time, respectively, across evaluation, follow-up, and discharge sessions. Bars indicate the percentage of respondents within each preference group selecting each reason. Multiple responses were allowed; therefore, percentages were calculated within each preference group and do not sum to 100%.

Furthermore, among therapists who preferred longer discharge sessions (*n* = 59), the most commonly reported reasons included providing more time for patient education (69%), physical examination (51%), and exercise prescription or monitoring (49%). Other reasons were explaining the treatment plan (41%), completing documentation (41%), providing passive treatments (31%), preparing the clinic or performing cleanup (24%), allowing time to change clothes (22%), warming up (19%), and obtaining patient history (20%). A few therapists (7%) also noted other reasons, such as facilitating family-centered care, conducting reevaluations, or performing outcome measures. However, therapists who preferred shorter discharge sessions (*n* = 34) primarily reported avoiding wasted time (71%), followed by a desire to see more patients (41%) and reduce working hours (15%). Data are illustrated in Fig. 6.

## Discussion

This study aimed to explore physical therapy session durations across evaluation, follow-up, and discharge sessions in outpatient settings, while also examining therapists’ satisfaction on adequacy and perceived optimal duration. Across all session types, the most frequently reported durations were 15–25 min and 30–40 min.

Satisfaction with current durations exceeded 50% across most session types, though it varied based on patient population, daily caseload, and overbooking status. Therapists who desired longer sessions most often reported the need for more time to conduct physical examinations, prescribe and monitor exercises, or deliver patient education. A smaller proportion preferred shorter sessions, to avoid wasting time, and to see more patients. The most commonly preferred session durations were 45–55 min for evaluation sessions and 30–40 min for both follow-up and discharge sessions.

Session duration varied clearly across patient populations in our findings, with longer durations more frequently reported in pediatric and neurological cases compared to musculoskeletal and sports settings. This pattern was consistent across evaluation, follow-up, and discharge sessions. These differences can be a reflection of the specific clinical demands associated with each population. Neurological rehabilitation often requires task-specific, repetitive, and individualized interventions such as gait retraining, neuromotor re-education, and functional mobility training, which are inherently time consuming due to both patient needs and the complexity of delivery (Fritz et al., 2011; Rice et al., 2016; Paniagua-Monrobel et al., 2023). Similarly, pediatric physical therapy frequently involves multifaceted approaches including parent education, play-based motor learning, behavioral engagement, and coordination with interdisciplinary teams, all of which extend the time required for effective sessions (Chavan & Sharath, 2023; Rapport et al., 2014; Ruiz-González et al., 2019). Sports rehabilitation demonstrated intermediate session durations in our data. Sports-focused sessions frequently incorporate structured functional assessments, performance monitoring, and return-to-sport protocols that are more goal-oriented and criteria-driven than general musculoskeletal care. These interventions, such as hop testing, isokinetic strength evaluation, and phased rehabilitation planning, are typically standardized and efficiently delivered, though they still require clinical precision and progression monitoring (Harris et al., 2014; Lorenz & Morrison, 2015; Hysing‐Dahl et al., 2023). In contrast, musculoskeletal physical therapy sessions tend to be shorter, as shown in our data. These sessions often prioritize focused manual therapy, therapeutic exercise, and symptom monitoring, which can typically be delivered within shorter, more predictable timeframes without compromising care quality (Waters et al., 2016; O’Keeffe et al., 2015). Taken together, these patterns suggest that session duration is shaped by the nature and complexity of clinical interventions required by each patient group. This has important implications for resource allocation and scheduling policies, emphasizing the need for population-specific planning that aligns clinical time with care demands.

Overbooking is commonly used in outpatient settings to mitigate no-shows and improve scheduling efficiency (Zhe-cheng, Hoon & Liang, 2012; Xie, Fan & Zhong, 2022; Huang & Zuniga, 2012; LaGanga & Lawrence, 2007). While such strategies can enhance resource utilization, they may also introduce unintended consequences, including increased wait times and provider workload (LaGanga & Lawrence, 2007). Our findings indicate that physical therapists who practice overbooking consistently reported higher dissatisfaction with session duration across all session types. This trend was evident in shorter sessions, particularly 15–25 min follow-up sessions, where dissatisfaction among overbooked therapists reached 38%, compared to 18% among those not overbooking. Even in mid-range durations such as 45–55 min, satisfaction remained lower in the overbooking group. These results indicate that overbooking was observed alongside lower therapist satisfaction, particularly in shorter session durations within outpatient physical therapy.

Therapists’ satisfaction with session duration also varied based on daily caseload. Those seeing fewer patients per day (1–5) reported the highest satisfaction across all session types, while satisfaction declined as caseload increased, reaching its lowest in the group treating 16 or more patients daily. This pattern suggests that higher caseloads coincided with lower satisfaction levels. Previous studies have similarly shown that higher patient loads are associated with increased time pressure, reduced care quality, and lower job satisfaction, and were major contributors to workplace stress among physical therapists (Fisher et al., 2012; Lindsay et al., 2008). Additionally, concerns have been raised that higher volumes and reduced face time with patients may jeopardize both therapist and patient satisfaction, particularly when cost-cutting pressures and ‘care extenders’ are involved (Beattie et al., 2002). Accordingly, optimizing therapist caseloads may be essential to support satisfaction and ensure the delivery of high-quality care.

A minority of physical therapists reported a need for longer sessions. Among them, the most frequently selected optimal durations across all session types were 30–40 min and 45–55 min. Conversely, therapists who preferred shorter sessions most often selected 15–25 min. The most selected reasons for wanting longer sessions included more time for physical examination during evaluation visits, more time for exercise prescription and monitoring during follow-ups, and more time for patient education during discharge. This pattern suggests that time constraints may be perceived as limiting the ability to perform key components of care. The consistency of these task-specific concerns across session types underscores the importance of aligning session length with the functional demands of each visit.

Physical examination is a foundational component of physical therapy practice, essential for accurate assessment, diagnosis, and treatment planning. According to the World Confederation for Physical Therapy (World Physiotherapy, 2023), it involves systematic evaluation of posture, movement, function, and physical impairments to guide clinical decision-making (World Physiotherapy, 2023). This process requires adequate time to be performed thoroughly (Gelisanga, Ocden & Angeles, 2024). When time is limited, therapists may be unable to complete all necessary assessment steps, which could explain their desire for longer session durations.

Exercise prescription and monitoring are essential physical therapy interventions supported by a strong body of evidence across rehabilitation disciplines (Smith, Snodgrass & Osmotherly, 2025). Proper exercise delivery requires time to instruct, observe, correct, and adjust techniques. Inadequate session time during follow-up visits may disrupt this process, contributing to therapists’ reported need for more time. Similarly, patient education should be individualized, evidence-based, and integrated with other active interventions. Core content may include advice on the importance of physical activity, strategies to remain active, and condition-specific self-management (George et al., 2021). While patient education is a recommended component of care, its quality depends on having sufficient time for explanation, discussion, and engagement (George et al., 2021). Without this, educational content may risk being rushed or deprioritized. Other patient-related factors also warrant consideration. Spiteri et al. (2022) found that cognitive status and fatigue were significantly associated with variation in session duration. This suggests that even when session durations are fixed, the ability to complete key tasks such as: physical examination, exercise monitoring, or patient education may still be limited.

Therapists who preferred shorter sessions often reported the desire to see more patients or avoid wasting time. This may reflect a belief that effective care can still be delivered in less time, or a need to accommodate clinic volume, institutional policies, or caseload-based compensation structures.

Taken together, patterns in this sample, in which shorter session durations and higher daily caseloads/overbooking coincided with lower satisfaction, may reflect a mismatch between institutional expectations for throughput and the therapist’s expectations for the clinical encounter. Productivity standards and high-volume scheduling have been linked to lower job satisfaction in physical therapists (Berry et al., 2022; Patel & Bartholomew, 2021), indicating that scheduling policies are not only operational decisions but also clinical constraints that shape each encounter. Limiting the available time for assessment, exercise supervision, and patient education may, in practice, restrict the therapist’s clinical autonomy during the session by reducing the opportunity to apply independent clinical judgment in the patient’s best interest, which is described as a professional and ethical responsibility in physical therapy (American Physical Therapy Association, 2025c). These activities are considered core components of effective physiotherapy and patient participation in care (HIOW Healthcare (NHS), 2025; Hush, Cameron & Mackey, 2011; Smith, Snodgrass & Osmotherly, 2025).

Within our descriptive scope, these distributions can serve as scheduling benchmarks by setting default session duration ranges by visit phase and patient population, and by reviewing overbooking and daily caseload targets in comparable services. This can orient clinical practice and managerial decisions on service organization and policy.

This study offers valuable insights into the relatively underexplored topic of session duration satisfaction and preferences among physical therapists. A notable strength is the large sample size, which enhances the robustness of the findings. However, several limitations should be acknowledged. The study relied on self-reported data, which may be subject to recall bias or varying personal interpretations of session timing. Additionally, the survey was distributed online and in English, potentially limiting participation to therapists with internet access and English proficiency, and the language of the questionnaire may have posed a barrier that influenced how some respondents interpreted or answered certain questions. The use of convenience sampling and the high proportion of participants with fewer years of experience may limit the generalizability of the findings; an overall response rate could not be calculated, and non-response bias cannot be excluded, and regional participation may be uneven, which could affect representativeness. The instrument was developed for this study and refined after a small pilot; no formal validation or reliability testing was performed. Future research should incorporate more objective measures, such as direct observation or time tracking, to validate reported session durations and examine their relationship with clinical outcomes. Longitudinal studies could also assess how changes in scheduling models affect therapist satisfaction, patient outcomes, and clinic efficiency over time.

## Conclusions

This study provides the first overview of outpatient physical therapy session durations across multiple patient populations and session types. The most frequently reported durations were 15–25 min and 30–40 min. Most therapists reported high satisfaction with their current session durations. Nonetheless, a clear trend emerged in which overbooking and higher daily caseloads were observed alongside lower therapist satisfaction. These patterns warrant further investigation to better understand their impact on clinical practice. The findings offer a foundation for developing policies and regulations concerning session durations in outpatient physical therapy. Future research should explore how session duration affects clinical outcomes to further optimize care delivery in this setting.

## Supplemental Information

10.7717/peerj.21394/supp-1 Supplemental Information 1 Raw Data.

10.7717/peerj.21394/supp-2 Supplemental Information 2 CodeBook.

10.7717/peerj.21394/supp-3 Supplemental Information 3 Questionnaire.

## Additional Information and Declarations

### Data Availability

The following information was supplied regarding data availability:

The raw data is available in the Supplemental Files.
