# Supplementary material for: From evaluation to discharge: a cross-sectional study of therapist satisfaction with session duration in outpatient physical therapy
Source: PeerJ. 2026 Jun 8;14:e21394. doi: 10.7717/peerj.21394 (PMC13256120; doi:10.7717/peerj.21394)
Supplement: Supplemental Information 2 [file peerj-14-21394-s002.docx]

| **Variable (Column)** | **Values** |
| --- | --- |
| Do you want to participate in this study? | 1=Yes, I agree to participate in this study and I know that my response will be completely anonymous., 2=No, I don’t agree to participate in the study. |
| What is your sex? | 1=Male, 2=Female |
| What is your nationality? | 1=Saudi, 2=Non-Saudi |
| How old are you? | 1=20-24, 2=25-29, 3=30-34, 4=35-39, 5=40-44, 6=45-49, 7=50-54, 8=55-59, 9=60+ |
| Which province do you work at? | 1=Riyadh Province, 2=Mecca Province, 3=Eastern Province, 4=Al-Qassim Province, 5=Medina Province, 6=Hail Province, 7=Tabuk Province, 8=Northern Border Province, 9=Najran Province, 10=Jazan Province, 11=Al-Jawf Province, 12=Al-Bahah Province, 13=Asir Province |
| Primary patient population seen by the therapist. | 1=Musculoskeletal, 2=Orthopedic, 3=Neurology, 4=Cardiopulmonary And Vascular, 5=Sports, 6=Pediatric, 7=Women's Health, 8=Other |
| What is your highest level of education? | 1=Bachelor of Science (BSc), 2=Master of Science (MSc), 3=Doctor of Philosophy (PhD) |
| What is your work setting? (the majority of your work) | 1=Outpatients department in a public hospital, 2=Outpatients department in a private hospital, 3=Outpatient's private clinic, 4=Outpatient's public clinic, 5=Inpatient department in a public hospital, 6=Inpatient department in a private hospital, 7=Homecare, 8=Educational Clinic, 9=Other |
| How many years of experience do you have? | 1-21+ |
| On an average, how many patients do you see daily? | 1-21+ |
| Do you regularly see more than a patient in a single session separately? (not group sessions) | 1=Yes, 2=No |
| What is the current duration of the evaluation session at your workplace? OUT | 1=15-25 minutes, 2=30-40 minutes, 3=45-55 minutes, 4=60-70 minutes, 5=75-85 minutes, 6=90-100 minutes, 7=105-115 minutes, 8=120+ minutes |
| Are you satisfied with your current evaluation session duration? OUT | 1=Yes, 2=No, 3=Neutral |
| Do you want more or less time in your current evaluation session duration? OUT | 1=I want more time, 2=I want less time, 3=I believe my current session duration is optimal. |
| What do you think the optimal duration of the evaluation session is? (Want more) OUT | 1=15-25 minutes, 2=30-40 minutes, 3=45-55 minutes, 4=60-70 minutes, 5=75-85 minutes, 6=90-100 minutes, 7=105-115 minutes, 8=120+ minutes |
| Why do you want more time for your evaluation session? OUT | 1=To allow more time for history taking., 2=To allow more time for physical examination., 3=To allow more time for passive treatment (i.e modalities, manual therapy, etc...)., 4=To allow more time to explain the treatment plan., 5=To allow more time for patient education., 6=To allow more time for exercise prescription/monitoring., 7=To allow more time for documentation., 8=To allow more time for patients to warm-up., 9=To allow more time for patient to change clothes and get ready., 10=To allow more time for treatment area preparation and cleanup., 11=Other |
| What do you think the optimal duration of the evaluation session is? (Want less) OUT | 1=15-25 minutes, 2=30-40 minutes, 3=45-55 minutes, 4=60-70 minutes, 5=75-85 minutes, 6=90-100 minutes, 7=105-115 minutes, 8=120+ minutes |
| Why do you want less time for your evaluation session? OUT | 1=To see more patients., 2=To avoid wasting therapist and patient time., 3=To cut working hours., 4=Other |
| What is the current duration of the follow-up session at your workplace? OUT | 1=15-25 minutes, 2=30-40 minutes, 3=45-55 minutes, 4=60-70 minutes, 5=75-85 minutes, 6=90-100 minutes, 7=105-115 minutes, 8=120+ minutes |
| Are you satisfied with your current follow-up session duration? OUT | 1=Yes, 2=No, 3=Neutral |
| Do you want more or less time in your current follow-up session duration? OUT | 1=I want more time, 2=I want less time, 3=I believe my current session duration is optimal. |
| What do you think the optimal duration of the follow-up session is? (Want more) OUT | 1=15-25 minutes, 2=30-40 minutes, 3=45-55 minutes, 4=60-70 minutes, 5=75-85 minutes, 6=90-100 minutes, 7=105-115 minutes, 8=120+ minutes |
| Why do you want more time for your follow-up session? OUT | 1=To allow more time for history taking., 2=To allow more time for physical examination., 3=To allow more time for passive treatment (i.e modalities, manual therapy, etc...)., 4=To allow more time to explain the treatment plan., 5=To allow more time for patient education., 6=To allow more time for exercise prescription/monitoring., 7=To allow more time for documentation., 8=To allow more time for patients to warm-up., 9=To allow more time for patient to change clothes and get ready., 10=To allow more time for treatment area preparation and cleanup., 11=Other |
| What do you think the optimal duration of the follow-up session is? (Want less) OUT | 1=15-25 minutes, 2=30-40 minutes, 3=45-55 minutes, 4=60-70 minutes, 5=75-85 minutes, 6=90-100 minutes, 7=105-115 minutes, 8=120+ minutes |
| Why do you want less time for your follow-up session? OUT | 1=To see more patients., 2=To avoid wasting therapist and patient time., 3=To cut working hours., 4=Other |
| What is the current duration of the discharge (last) session at your workplace? OUT | 1=15-25 minutes, 2=30-40 minutes, 3=45-55 minutes, 4=60-70 minutes, 5=75-85 minutes, 6=90-100 minutes, 7=105-115 minutes, 8=120+ minutes |
| Are you satisfied with your current discharge (last) session duration? OUT | 1=Yes, 2=No, 3=Neutral |
| Do you want more or less time in your current discharge (last) session duration? OUT | 1=I want more time, 2=I want less time, 3=I believe my current session duration is optimal. |
| What do you think the optimal duration of the discharge (last) session is? (Want more) OUT | 1=15-25 minutes, 2=30-40 minutes, 3=45-55 minutes, 4=60-70 minutes, 5=75-85 minutes, 6=90-100 minutes, 7=105-115 minutes, 8=120+ minutes |
| Why do you want more time for your discharge (last) session? OUT | 1=To allow more time for history taking., 2=To allow more time for physical examination., 3=To allow more time for passive treatment (i.e modalities, manual therapy, etc...)., 4=To allow more time to explain the treatment plan., 5=To allow more time for patient education., 6=To allow more time for exercise prescription/monitoring., 7=To allow more time for documentation., 8=To allow more time for patients to warm-up., 9=To allow more time for patient to change clothes and get ready., 10=To allow more time for treatment area preparation and cleanup., 11=Other |
| What do you think the optimal duration of the discharge (last) session is? (Want less) | 1=15-25 minutes, 2=30-40 minutes, 3=45-55 minutes, 4=60-70 minutes, 5=75-85 minutes, 6=90-100 minutes, 7=105-115 minutes, 8=120+ minutes |
| Why do you want less time for your discharge (last) session? OUT | 1=To see more patients., 2=To avoid wasting therapist and patient time., 3=To cut working hours., 4=Other |
