# Supplementary material for: From evaluation to discharge: a cross-sectional study of therapist satisfaction with session duration in outpatient physical therapy
Source: PeerJ. 2026 Jun 8;14:e21394. doi: 10.7717/peerj.21394 (PMC13256120; doi:10.7717/peerj.21394)
Supplement: Supplemental Information 3 [file peerj-14-21394-s003.pdf]

Dear ***Participant***,

You are invited to participate in this survey and your contribution is greatly valued as it will help us to achieve the intended purpose of this study.

The purpose of this online survey is to investigate the opinion of physical therapists working in Saudi Arabia about the optimal session duration. It will take approximately 6-8 minutes of your time to be completed. Your participation is completely voluntary and you can withdraw at any time.

The responses are completely anonymous and confidential
and researchers will not be able to identify participants. You will not be asked for your name or any other identifying information. We will use this collected information for the research purpose only. (KSU-IRB #E-24-8553)

Please feel free to call Mr. Abdulaziz Aljumaah, with Mobile No. +966 565133000 to answer your question

Do you want to participate in this study? *

Mark only one oval.

- Yes, I agree to participate in this study and I know that my response will be completely anonymous.
- No, I don't agree to participate in the study.

Physical Therapist Information

In this section you will answer some demographic questions.

1. What is your sex? *

Mark only one oval.

- ⬭ Male
- ⬭ Female

2. What is your nationality? *

Mark only one oval.

- ⬭ Saudi
- ⬭ Non-Saudi

3. How old are you?

*

Dropdown

Mark only one oval.

- 20-24
- 25-29
- 30-34
- 35-39
- 40-44
- 45-49
- 50-54
- 55-59
- 60+

4. Which province do you work at?

*

Dropdown

Mark only one oval.

- Riyadh Province
- Mecca Province
- Eastern Province
- Al-Qassim Province
- Medina Province
- Hail Province
- Tabuk Province
- Northern Border Province
- Najran Province
- Jazan Province
- Al-Jawf Province
- Al-Bahah Province
- Asir Province

5. Primary patient population seen by the therapist. *

Mark only one oval.

- Musculoskeletal
- Orthopedic
- Neurology
- Cardiopulmonary And Vascular
- Sports
- Pediatric
- Women's Health
- Other: ____________________

6. What is your highest level of education? *

Mark only one oval.

- Bachelor of Science (BSc)
- Master of Science (MSc)
- Doctor of Philosophy (PhD)

7. What is your work setting? (the majority of your work) *

Mark only one oval.

- ⬭ Outpatients department in a public hospital
- ⬭ Outpatients department in a private hospital
- ⬭ Outpatient's private clinic
- ⬭ Outpatient's public clinic
- ⬭ Inpatient department in a public hospital
- ⬭ Inpatient department in a private hospital
- ⬭ Homecare
- ⬭ Educational Clinic
- ⬭ Other: ______________________

8. How many years of experience do you have? *

Dropdown

Mark only one oval.

- 1
- 2
- 3
- 4
- 5
- 6
- 7
- 8
- 9
- 10
- 11
- 12
- 13
- 14
- 15
- 16
- 17
- 18
- 19
- 20
- 21+

9. On an average, how many patients do you see daily? * Dropdown

Mark only one oval.

- 1
- 2
- 3
- 4
- 5
- 6
- 7
- 8
- 9
- 10
- 11
- 12
- 13
- 14
- 15
- 16
- 17
- 18
- 19
- 20
- 21+

10. Do you regularly see more than a patient in a single session Separately? (not group sessions) *

Mark only one oval.

- Yes
- No

Current Evaluation Session

In this section you will answer questions regarding your evaluation session, which is the first session for a new patient. (Not triage)

11. What is the **current duration** of the evaluation session at your workplace? *

(on average)

Mark only one oval.

- 15-25 minutes
- 30-40 minutes
- 45-55 minutes
- 60-70 minutes
- 75-85 minutes
- 90-100 minutes
- 105-115 minutes
- 120+ minutes

12. Are you **satisfied** with your current evaluation session duration? *

Mark only one oval.

- Yes
- No
- Neutral

13. Do you want **more or less** time in your current evaluation session duration? *

Mark only one oval.

- I want more time *Skip to question 14*

- [ ] I want less time *Skip to question 16*
- [ ] I believe my current session duration is optimal. *Skip to question 18*

Optimal Evaluation Session Duration

In this section you will answer questions regarding the optimal evaluation session duration, which is the first session for a new patient. (Not triage)

14. What do you think the **optimal duration** of the evaluation session is? * (on average)

Mark only one oval.

- [ ] 15-25 minutes
- [ ] 30-40 minutes
- [ ] 45-55 minutes
- [ ] 60-70 minutes
- [ ] 75-85 minutes
- [ ] 90-100 minutes
- [ ] 105-115 minutes
- [ ] 120+ minutes

15. Why do you want **more time** for your evaluation session? (You can choose multiple options) *

Tick all that apply.

- [ ] To allow more time for history taking.
- [ ] To allow more time for physical examination.
- [ ] To allow more time for passive treatment (i.e modalities, manual therapy, etc...).
- [ ] To allow more time to explain the treatment plan.
- [ ] To allow more time for patient education.
- [ ] To allow more time for exercise prescription/monitoring.
- [ ] To allow more time for documentation.
- [ ] To allow more time for patients to warm-up.
- [ ] To allow more time for patient to change clothes and get ready.
- [ ] To allow more time for treatment area preparation and cleanup.
- [ ] Other: ______________________

Skip to question 18

Optimal Evaluation Session Duration

In this section you will answer questions regarding the optimal evaluation session duration, which is the first session for a new patient. (Not triage)

16. What do you think the **optimal** duration of the evaluation session is? *

(on average)

Mark only one oval.

- ( ) 15-25 minutes
- ( ) 30-40 minutes
- ( ) 45-55 minutes
- ( ) 60-70 minutes
- ( ) 75-85 minutes
- ( ) 90-100 minutes
- ( ) 105-115 minutes
- ( ) 120+ minutes

17. Why do you want **less time** for your evaluation session? (You can choose multiple options) *

Tick all that apply.

- [ ] To see more patients.
- [ ] To avoid wasting therapist and patient time.
- [ ] To cut working hours.
- [ ] Other: ____________________

Skip to question 18

Current Follow-up Session

In this section you will answer questions regarding the follow-up session.

18. What is the **current duration** of the follow-up session at your workplace? *

(on average)

Mark only one oval.

- 15-25 minutes
- 30-40 minutes
- 45-55 minutes
- 60-70 minutes
- 75-85 minutes
- 90-100 minutes
- 105-115 minutes
- 120+ minutes

19. Are you **satisfied** with your current follow-up session duration? *

Mark only one oval.

- Yes
- No
- Neutral

20. Do you want **more or less** time in your current follow-up session duration? *

Mark only one oval.

- I want more time *Skip to question 21*
- I want less time *Skip to question 23*
- I believe my current session duration is optimal. *Skip to question 25*

Optimal Follow-up Session Duration

In this section you will answer questions regarding the optimal follow-up session duration.

21. What do you think the **optimal duration** of the follow-up session is? *
(on average)

Mark only one oval.

- 15-25 minutes
- 30-40 minutes
- 45-55 minutes
- 60-70 minutes
- 75-85 minutes
- 90-100 minutes
- 105-115 minutes
- 120+ minutes

22. Why do you want **more time** for your follow-up session? (You can choose multiple options) *

Tick all that apply.

- To allow more time for history taking.
- To allow more time for physical examination.
- To allow more time for passive treatment (i.e modalities, manual therapy, etc...).
- To allow more time to explain the treatment plan.
- To allow more time for patient education.
- To allow more time for exercise prescription/monitoring.
- To allow more time for documentation.
- To allow more time for patients to warm-up.
- To allow more time for patient to change clothes and get ready.
- To allow more time for treatment area preparation and cleanup.
- Other: ______________________

Skip to question 25

Optimal Follow-up Session Duration

In this section you will answer questions regarding the optimal follow-up session duration.

23. What do you think the **optimal** duration of the follow-up session is? *

(on average)

Mark only one oval.

- 15-25 minutes
- 30-40 minutes
- 45-55 minutes
- 60-70 minutes
- 75-85 minutes
- 90-100 minutes
- 105-115 minutes
- 120+ minutes

24. Why do you want **less time** for your follow-up session? (You can choose multiple options) *

Tick all that apply.

- [ ] To see more patients.
- [ ] To avoid wasting therapist and patient time.
- [ ] To cut working hours.
- [ ] Other: ______________________

Skip to question 25

Current Discharge (Last) Session

In this section you will answer questions regarding the discharge session, which is the last

session of the patient.

25. What is the **current duration** of the discharge (last) session at your workplace? *

(on average)

Mark only one oval.

- 15-25 minutes
- 30-40 minutes
- 45-55 minutes
- 60-70 minutes
- 75-85 minutes
- 90-100 minutes
- 105-115 minutes
- 120+ minutes

26. Are you **satisfied** with your current discharge (last) session duration? *

Mark only one oval.

- Yes
- No
- Neutral

27. Do you want **more or less** time in your current discharge (last) session duration? *

Mark only one oval.

- I want more time *Skip to question 28*
- I want less time *Skip to question 30*
- I believe my current session duration is optimal. *Skip to question 32*

Optimal Discharge (Last) Session Duration

In this section you will answer questions regarding the optimal discharge session duration, which is the last session of the patient.

28. What do you think the **optimal duration** of the discharge (last) session is? *

Mark only one oval.

- ◯ 15-25 minutes
- ◯ 30-40 minutes
- ◯ 45-55 minutes
- ◯ 60-70 minutes
- ◯ 75-85 minutes
- ◯ 90-100 minutes
- ◯ 105-115 minutes
- ◯ 120+ minutes

29. Why do you want **more time** for your discharge (last) session? (You can choose multiple options) *

Tick all that apply.

- [ ] To allow more time for history taking.
- [ ] To allow more time for physical examination.
- [ ] To allow more time for passive treatment (i.e modalities, manual therapy, etc...).
- [ ] To allow more time to explain the treatment plan.
- [ ] To allow more time for patient education.
- [ ] To allow more time for exercise prescription/monitoring.
- [ ] To allow more time for documentation.
- [ ] To allow more time for patients to warm-up.
- [ ] To allow more time for patient to change clothes and get ready.
- [ ] To allow more time for treatment area preparation and cleanup.
- [ ] Other: ____________________

Skip to Section 11

Optimal Discharge (Last) Session Duration

In this section you will answer questions regarding the optimal discharge session duration, which is the last session of the patient.

30. What do you think the **optimal** duration of the discharge (last) session is? *

Mark only one oval.

- 15-25 minutes
- 30-40 minutes
- 45-55 minutes
- 60-70 minutes
- 75-85 minutes
- 90-100 minutes
- 105-115 minutes
- 120+ minutes

31. Why do you want **less time** for your discharge (last) session? (You can choose multiple options) *

Tick all that apply.

- [ ] To see more patients.
- [ ] To avoid wasting therapist and patient time.
- [ ] To cut working hours.
- [ ] Other: ____________________

Skip to Section 11

Please Press "Submit" below

Please press "Submit"

This content is neither created nor endorsed by Google.

Google Forms
